# Stakeholder Perceptions of Disease Management for Dairy Calves: “It’s Just Little Things That Make Such a Big Difference”

**DOI:** 10.3390/ani11102829

**Published:** 2021-09-28

**Authors:** Laura J. Palczynski, Emma C. L. Bleach, Marnie L. Brennan, Philip A. Robinson

**Affiliations:** 1 Department of Animal Health, Behaviour and Welfare, Harper Adams University, Newport TF10 8NB, Shropshire, UK; laurap@i4agri.org; 2 Arthur Rank Centre, Livestock Department, Innovation for Agriculture, Stoneleigh Park, Stoneleigh CV8 2LZ, Warwickshire, UK; 3 Department of Agriculture and Environment, Harper Adams University, Newport TF10 8NB, Shropshire, UK; ebleach@harper-adams.ac.uk; 4 Sutton Bonington Campus, School of Veterinary Medicine and Science, University of Nottingham, Leicestershire LE12 5RD, UK; Marnie.Brennan@nottingham.ac.uk

**Keywords:** dairy calf health, disease, stockmanship, stakeholder attitudes, interviews

## Abstract

**Simple Summary:**

The scientific research literature indicates that calf health and welfare could be improved on dairy farms globally. Preventing disease in calves or treating disease quickly and appropriately when necessary has been shown to improve lifelong health and production parameters, but success is ultimately determined by the persons responsible for calf care at the farm level. This paper presents findings from semi-structured interviews with farmers and advisors about dairy calf rearing in England. This research highlights the vital importance of the human element of calf rearing, and how it influences the interactions among calves, pathogens, and the environment to maintain calves in good health or otherwise. Calf rearers often found it difficult to pinpoint a specific disease problem, causal factor, or likely solution, causing frustration and a perceived inability to reduce calf morbidity and mortality, which resulted in inaction. The person(s) responsible for calf rearing are often not those who control farm finances, which can often result in underinvestment in facilities and stockmanship efforts hindered by suboptimal calf housing. It is, therefore, essential that efforts to promote disease management practices not only focus on technical solutions, but also the mindset, priorities, and experiences of the persons responsible for calf rearing and the allocation of farm finances.

**Abstract:**

Calf morbidity and mortality rates are often high in dairy herds, raising animal welfare concerns and negatively affecting farm economic efficiency and future performance. Disease prevention is critical to maintain calves in good health, but interventions are dependent upon the persons conducting them. This paper explores the perceptions of farmers, farm workers, veterinarians, and other advisors on the management of calfhood disease on dairy farms in England. Participants were recruited using purposive and “snowball” sampling, resulting in 40 in-depth, semi-structured interviews—26 with dairy farmers and 14 with advisors. Interviews were recorded, transcribed, and thematically coded. Three major themes were derived on the basis of interview data: disease occurrence and treatments, management of calf environment, and the role of stockmanship and perceived control. Respiratory and gastrointestinal diseases in calves were those reported to be most problematic on dairy farms. Limited time and financial resources caused some farmers and advisors to experience a perceived inability to control calf health without antimicrobial treatments. Overall, the findings emphasise the importance of human influences on calf health and disease in the context of influencing the interactions among the host, pathogens, and the environment. Further research should investigate what “attention to detail” means within different farm contexts and practices, as this was believed to be important in the promotion of better husbandry standards and health. We recommend the use of supportive knowledge exchange processes, including facilitation, to empower farmers to promote continuous improvement in calf health.

## 1. Introduction

Calf morbidity and mortality rates are often high in dairy herds worldwide, raising animal welfare concerns and negatively affecting farm economic efficiency [1,2,3]. Contagious disease, particularly bovine respiratory disease (BRD) and diarrhoea (commonly referred to as pneumonia and scour, respectively), is the leading cause of mortality in calves under 6 months of age [4,5], with some farms experiencing disease incidences of over 70% [6,7]. Costs relating to health and disease amount to an estimated 4.1% of overall rearing costs from birth to first calving, and they represent 9.6% of costs in the preweaning period, with the mean cost of heifer mortality calculated at approximately 140 GBP (198 USD) per surviving heifer [1]. Furthermore, calfhood disease negatively impacts first lactation performance [8], and heifers treated for pneumonia as calves have shorter longevity than their cohorts [2]. The total lifetime cost of respiratory disease has been estimated as 772 GBP (1089 USD) for dairy heifers in the United Kingdom (UK) [9].

Accurate data regarding calf disease incidence are lacking, in part because of poor recording on farms [4,10], but also because of difficulties regarding the diagnosis of disease [4]. There are multiple causal pathogens for BRD and diarrhoea, including viruses, protozoa, and bacteria, as well as non-infectious causes such as those relating to feeding management. *Cryptosporidium parvum*, coronavirus, and rotavirus are the most common pathogens causing enteric disease in calves [4,6,11]. BRD is caused by corona- and para-influenza viruses, *Pasteurella* spp., and *Mycoplasma* spp., amongst others, but it is comparatively more challenging to determine the causal agent for respiratory disease than for diarrhoea [4]. Additionally, bovine viral diarrhoea (BVD) is a costly disease [12], which negatively affects the productivity and immune function of affected animals [13], making calves susceptible to secondary infections from enteric and respiratory pathogens [14]; however, efforts are being made to tackle the disease in England, which should have a positive impact on calf health. BVDFree England (2020) [15] is a voluntary scheme which aims to eliminate BVD from all cattle in England by 2022, primarily using diagnostic testing [13,16]—either by administering an antibody blood test on a sample of unvaccinated youngstock at 9–18 months of age or by conducting “tag and test” or blood samples on all calves born to detect BVD antigen or antibodies to the virus [15].

Antibiotic use in calves is an important consideration for antimicrobial stewardship, but treatments can be under-recognised in farm usage metrics, largely due to the smaller liveweight of calves compared to adults [17]. Standard treatment for diarrhoea at the farm level should consist of oral rehydration therapy, continuation of milk feeding, and antimicrobial treatments only when appropriate [18,19]. The administration of nonsteroidal anti-inflammatory drugs (NSAIDs) also relieves symptoms, improving weight gain and feed intakes [20,21,22]. Treatment for pneumonia generally involves appropriate antibiotic treatment and NSAIDs [23], although antibiotics may not be necessary in all cases [22,24]. Disease prevention is critical to reduce disease incidence, antibiotic use, the need for interventions [25], and their associated costs, as well as to maintain calves in good health to achieve target growth performance and more positive welfare. Furthermore, antibiotic usage is perceived to be greatest during the calf rearing period of cattle production systems; thus, maintaining calves in good health will contribute to reduction targets aimed at combating antimicrobial resistance [26].

The epidemiological triad describes how infectious disease is dependent upon interactions among the host (calf), agent (pathogens), and environment [27]. Therefore, disease control measures must include supporting calves’ immune systems through good colostrum management, suitable nutrition, and vaccination, whilst also controlling pathogen load and challenge in the environment through good hygiene practices and biosecurity measures [28], alongside adequate ventilation and drainage within calf housing [23,29,30]. Management of groups of calves is also key; although the effects of individual compared to group housing on calf health are unclear due to conflicting findings in the literature [23,31,32], group housing is considered better for growth and welfare [31]. Maintaining appropriate stocking rates and avoiding shared air spaces with older animals are also important to prevent spread of disease [30]. However, farms might struggle to achieve this as space restrictions or layout of existing farm buildings might limit their ability to accommodate calves appropriately. Additional pressures occur when farms are rearing more calves to increase herd size or are unable to offload surplus calves [33], particularly during bovine tuberculosis (bTB) outbreaks when movement and sales restrictions apply to animals from affected herds [34].

Achieving high standards for disease control is ultimately dependent on the persons responsible for planning and ensuring preventative measures are implemented—particularly the farmers, stockpersons, and veterinarians [35,36,37]. Individual values and priorities affect decision making [38,39], as does awareness of a problem and willingness to change practices, alongside perceived control over the situation and ability to make improvements [40,41]. Time, labour, and financial constraints can also pose barriers to making improvements to protect animal health and welfare on farm [42,43]. In addition, inconsistent information and advice can impinge on effective decisions and actions at the farm level, such as those relating to milk feeding of calves [44]. It is, therefore, very important to understand the personal and practical factors contributing to disease management.

This paper focuses on the attitudes and decision making of farmers, farm workers, veterinarians, and other farm advisors such as nutritionists and pharmaceutical industry representatives related to the management of calfhood disease on dairy farms in England. Using a qualitative interview-based approach, the study sought to uncover the opinions and experiences of these key stakeholders. The primary focus of this paper was not to quantify which infectious diseases calves suffered from and how frequently, nor to determine specific treatment protocols; rather, the methodology aimed to uncover and reflect upon the most pressing concerns and priorities of the participants to gain an understanding of the wider context and issues surrounding disease management in dairy calf enterprises. Similar approaches have been used to investigate, for example, perspectives regarding calf management before and after benchmarking reports [45], calf mortality rates [40], and calf welfare in organic systems [46]. It should also be noted that the same participants’ views on the related subjects of colostrum management and feeding, which also impact calf husbandry and health, are not covered in this paper as they have previously been published elsewhere [43,44].

It is increasingly recognised that research methodologies from the social sciences provide valuable insights into issues concerning animal health and welfare [47,48]. The views and rationales of key stakeholders need to be better understood to understand why scientific research findings and recommended best practice may or may not be implemented on dairy farms, as well as to help find applied solutions that enhance animal welfare and farm profitability [49,50].

## 2. Materials and Methods

The research presented in this paper was based on a critical realist paradigm, meaning that subjective experiences and beliefs were deemed as valid as objective facts to help understand phenomena in more depth [51]. In-depth, semi-structured interviews followed by thematic analysis of the interview data were used to achieve a holistic understanding of calf management on English dairy farms including colostrum management [43], calf feeding [44], and the perceived value of youngstock, advice, and calf performance monitoring [52]. This paper examines findings particularly related to disease management in dairy calves derived from the wider research study, and further details of the interview participants and their dairy herds can be found in the associated published papers [43,44].

The research was conducted and presented in line with Consolidated Criteria for Reporting Qualitative Research (COREQ) [53]. All participants gave their informed consent for interviews to be conducted, audio-recorded, transcribed, and securely stored, as well as for anonymised interview excerpts to be published. The study was conducted in accordance with the Declaration of Helsinki, and the protocol was approved under project number 75-201511 by the Harper Adams University Research Ethics Committee on 13 January 2016.

### 2.1. Data Collection—Interviews and Participants

Forty face-to-face semi-structured interviews (26 with farmers on 26 different farms, 14 with advisors) were conducted between May 2016 and June 2017 by the first author. The average interview length was 56 min, with a range of 26 to 90 min duration. Three interview formats were used: individual interviews in a seated setting (*n* = 23), joint interviews where two to three participants (*n* = 20) were interviewed together (*n* = 9), or walking interviews during a tour of the farm and calf facilities (*n* = 8). Questions used in the interviews were based on a topic guide and were deliberately broad, looking to obtain a general overview of participants’ views and experiences related to calf rearing on dairy farms, as well as to allow them to lead the discussion in the direction of their choice and on issues which mattered to them. Interviews were audio-recorded on a digital voice recorder and assigned a representative code, i.e., a letter referring to the type of participant (farmer, F; veterinarian, V; feed consultant, N; pharmaceutical company representative, DR; veterinary government advisor, GA), and numbered in chronological order for each grouping (F1, F2, F3, etc.).

Participants were recruited using non-random purposive and “snowball” sampling [54], starting with existing networks and contact with local veterinary practices, meeting individuals attending dairy events and conferences, and approaching persons suggested by interviewees. The primary aim was to recruit a wide selection of stakeholders involved directly in calf rearing and veterinarians and other professional advisors who provided expert advice on dairy farms and who were willing to engage in a discussion about dairy calf rearing. Participants were assured of anonymity, and all gave their written consent to be involved in the study. This recruitment method resulted in a variety of dairy production systems and herd sizes being represented. Three geographical areas in England were covered: the Southwest and Midlands (both high densities of dairy farms) and the Northeast (less intensive dairy focus). Interviewees included 37 dairy farmers (farm managers (*n* = 17), farm workers (*n* = 9), calf rearers (*n* = 8), and herd managers (*n* = 3)) and 14 advisors (veterinarians (*n* = 11), feed consultants (*n* = 2), and a veterinary pharmaceutical company representative (*n* = 1)). This variation satisfied the need for rich, detailed data from a range of contexts, in line with quality criteria for qualitative research [55]. Participants were considered “the expert” and were asked to confer their knowledge and experience to the researcher through the interview discussions.

### 2.2. Data Analysis—Thematic Analysis

Data collection and analysis were conducted using an iterative approach until it was judged that no new themes were emerging, indicating thematic saturation [56].

Audio recordings of the interviews were manually transcribed using f4transkript software (Version 6.2.5 Edu, audiotranskription.de, Marburg, Germany). Interview transcripts were thematically coded in NVivo for Windows (Version 11.4.1.1064 Pro, QSR International Pty Ltd., Doncaster, VIC, Australia) to group common extracts into themes [57]. Coding was conducted by the first author. First, content coding was used to group extracts according to topic [56], i.e., management practices, processes, and personal values. This helped to inform ongoing interviews and indicate focal topics for further analysis. Once data collection was completed, coding was repeated for in-depth exploration of extracts relating to each focal topic.

Extracts were chosen to represent the perceptions of participants which informed the construction of themes and explanations by the first author. The extracts most relevant to calf disease and its management tended to be in response to questions such as the following: “What are the main problems you experience regarding calves?” “What is important for successful calf rearing?” “Which changes would you like to make, if any, to your calf management or facilities?” Additional probing questions were asked to gain further insight into the participant’s initial response. Quotes from participants are presented within quotation marks; ellipses indicate omission of text, and square brackets indicate clarifications from the authors.

## 3. Results

Three major themes were drawn from the interview data: disease occurrence and treatments, management of calf environment, and the role of stockmanship and perceived control. Each of these themes is considered in this section of the paper.

### 3.1. Disease Occurrence and Treatments

#### 3.1.1. Pneumonia, Diarrhoea, and Mortality

Farmers and veterinarians considered BRD and diarrhoea (scour) to be the main threats to calf health. However, on individual farms, it was generally perceived that one was more problematic or common than the other (Table S1). Most farmer participants perceived pneumonia to be a bigger problem than scour for their calves (Table S1), according to their recollection of issues rather than treatment records, but some veterinarians disagreed with this farmer perception, as demonstrated by the following contrasting opinions of a farm manager and a veterinarian:

“Scours we don’t get so much of a problem with. We could count the number of cases on one hand that we get like in 6 months, or even a year.”

F9, farm manager.

“I would say scour is by far the most common, but a lot of farmers don’t perceive it as a problem. They’ll know the ones that die because of it, but they’ll often massively underestimate how much of a problem it is.”

V11, youngstock veterinarian.

Advisors were concerned that farmers often failed to record calf data to accurately assess morbidity and mortality rates.

“The change in the Red Tractor standards [assurance scheme] that came in 3–4 years ago, when it’s one of the requirements to actually track your calf mortality, and I think a lot of people maybe didn’t even know that. I think it’s quite scary.”

DR1, pharmaceutical company veterinary advisor.

Aside from a few calf rearers (F2, husband and wife F26) who announced their mortality figure from the previous year to be zero, and a dairy bull calf rearer who stated that their mortality rate was 2–3% (F17), farmers in this study generally relied on memory to assess calf mortality and tended to perceive low numbers of calf deaths.

“Mortality rate’s really, really low. Might have about one a month or something, I don’t even know that it would be that. I think we were below double figures last year and the year before.”

F11, farm administrator (herd size approximately 400, all-year-round calving).

The long-term significance of calfhood illness on adult performance was mentioned by several farmers. Some joked that they were unsure why they nursed some very poorly calves back to health, but seemed proud that they had. Others felt it was often better to cull calves that would not recover sufficiently to perform well as adults; on a couple of farms, there was evidence that calfhood pneumonia resulted in long-term lung damage that negatively affected performance and welfare in later life.

“You could persevere with some—we put them to sleep. They’re just gonna be poor doers and I don’t think it’s worth wasting several thousand pounds on rearing them when they’re just probably gonna give us poor lactations and just be problems … I’d rather get rid of them after a few weeks.”

F24, farm manager.

“There’re a few cows … they’ve had pneumonia as calves and it’s just coming out now, maybe second lactation, and it really, really hurt them.”

F22, herd manager.

#### 3.1.2. Understanding of Disease Processes and Treatments

Farmer participants demonstrated a basic understanding of disease processes. Pneumonia problems were mainly attributed to poorly ventilated, overstocked buildings and winter weather. Little consideration was given to the causal pathogens for pneumonia (aside from some mentioning *Mycoplasma bovis* issues); farmers tended to refer to the infectious causes of diarrhoea, particularly coccidiosis, cryptosporidiosis, and rotavirus (Table S1). Scour outbreaks were perceived to be linked to calf management: hygiene, stressful periods, mixing of groups, colostrum management, and milk feeding. Participants also referred to nutritional scour which they considered resulted from increasing calf milk replacer feeding rate (particularly in accelerated feeding programmes when increasing to >900 g milk powder/day), or when seasonal grass quality changed the profile of whole milk being fed. Preventive measures to protect calves against pneumonia and scour included a range of practices such as colostrum management, providing adequate nutrition, vaccinations, good hygiene practices and group management, and improvements to calf accommodation (Table S1). Some participants blanket-treated calves with Halocur^®^ (halofuginone lactate, MSD Animal Health UK Ltd., Milton Keynes, UK) to prevent diarrhoea caused by *Cryptosporidium parvum* (Table S1).

However, preventive measures did not negate the need for disease treatments. The key treatment used for diarrhoea was oral rehydration therapy (Table S2), because “the dehydration will kill them more than anything else” (F6, calf rearer). Pneumonia was usually treated with antibiotics, and, although only one participant mentioned “how painful pneumonia is … brings a huge compromise to animal welfare” (V4, farm veterinarian), several farmers appreciated the benefits of analgesic treatments (Table S2) to improve calf wellbeing and recovery:

“What we’ve found works best [is] the Metacam [meloxicam, Boehringer Ingelheim Animal Health UK Ltd., Bracknell, UK] anti-inflammatory painkiller. Just gets the calf up on its feet. You get it up, you get it eating, you get it drinking again … feeling better in itself, regardless [of] whether the infection’s gone, you’ve got a lot more chance of him coming right.”

F17, farm worker on calf rearing unit.

#### 3.1.3. Antimicrobial Stewardship

Both veterinarian and farmer participants were cognisant of antimicrobial resistance and expressed concern regarding the use of blanket prophylactic antibiotic treatments being “accepted as normal” (V4, farm veterinarian) on farms. Indeed, some participants shared their experiences of engaging with farmers for whom prophylactic antibiotic use was a routine management protocol for their calves.

“I do the computer work for another farm down the road, and they just feed their calves blanket antibiotics in their milk because they have lots of problems with them and … that’s the easiest, quickest fix.”

F2, calf rearer.

“A very big calf rearer … he said … ‘I buy 2000 calves a year and we don’t worry about BVD … because we feed antibiotic milk powder for five days when they arrive on farm’ … We cannot be doing that as an industry; that is not acceptable practice … that kind of stuff really frustrates me.”

DR1, pharmaceutical company veterinary advisor. Concerns were not limited to certain individuals. One veterinarian (V4) criticised the inattention to antibiotic usage stemming from treatments against *Mycoplasma bovis* within the dairy industry.

“*Mycoplasma bovis* in the national dairy herd is a huge, huge problem. No one talks about it … It’s probably the single biggest cause of antibiotic usage in dairy replacements.”

V4, farm veterinarian.

Antimicrobial stewardship appeared suboptimal when it came to treating ill calves. Apparently, some perceived antibiotics as the most effective solution for calf health problems, regardless of causal pathogen, as reported by one veterinarian.

“Particularly the older generation, ‘Why do I want to give them electrolytes when I could give them a pill that works?’ And you’re going … ‘There’s no reason to give antibiotics to something with rotavirus.’ It’s really difficult. Sometimes you just have to let them carry on giving the pills, provided they do the other things that you want them to do as well … [they think] it’s only the pills that have worked and nothing else…”

V7, farm veterinarian.

Indeed, some farmer participants treated scouring calves with antibiotics, even when the cause of diarrhoea was not believed to be bacterial (Table S2). There were mixed feelings about the need to take faecal samples to diagnose the cause; treatment decisions were often based on previous diagnoses in efforts to intervene quickly.

“If we get any scouring calves, we’ll take a [faecal] sample and give it to the vets and they’ll test for what’s actually wrong with it, and then we’ll be injecting them with the right drug … the medicines are expensive, so we don’t want to be injecting them and not utilising the medicine, and we don’t want to use more antibiotics than we need to.”

F1, calf rearer.

“Nine times out of ten you know it’s gonna be [coccidiosis] so I just dose [the calf] anyway and they seem to clear up. Trouble is that … you take [faecal] samples to the vets and [they say] ‘Oh yeah, but it’s at a very low level, just see how it goes on’ … It goes on, then a week later it’s got even worse, and you think ‘Well, I could’ve treated that a week ago and it wouldn’t have been knocked back.’”

F14, calf rearer.

#### 3.1.4. The Importance of Early Treatment Interventions

It was widely accepted among participants that early treatment for calf illness led to higher survival rates and treatment success:

“With a calf, you have to be on it. They’re babies. If they’re not very well, the next day they’re nearly dying.”

F18, calf rearer.

“If you [detect illnesses] quickly, then [calves] respond much better to treatment than if you leave them until they’re really sick.”

F2, calf rearer.

An experienced designated calf rearer, whose time and priorities were focused on calves, was generally considered to allow for higher-quality calf husbandry and earlier detection of behavioural signs of illness in calves.

“Say a calf looks a little bit sick, maybe it’s got its ear down, or you just know them, they just look a little bit off colour. Someone else would walk in the shed and say ‘What are you worried about? That calf looks fine!’ But you know it’s not. Take its temperature!”

F18, calf rearer.

“There’s a couple of farms I can think of, where if the stockman who rears the calves goes away for the weekend and the farmer rears the calves, there’ll be two or three dead calves come Monday because he’s not fed them right, or he’s not spotted the signs early enough.”

V3, youngstock veterinarian.

Technology was reported as a tool to facilitate the detection of early signs of disease. Farmers using automated milk feeders used notifications of slow drinking or lower feed consumption by calves as an early indicator of calf illness. Some farmers were also considering investing in TempVerified Calf Tags^®^ (FeverTags, https://fevertags.com (accessed on 11 July 2021), Dallas, TX, USA), which flash in response to sustained high temperature.

“I’ve looked at these tags that flash a light at you if [the calf is] hot … I might do a trial on that; do a couple of pens, and you’re not allowed to treat them for antibiotic unless you see there’s a temperature for pneumonia. That could cut our antibiotic usage … They’re quite expensive … I wouldn’t do it unless it saves us money.”

F20, farm manager.

#### 3.1.5. Vaccines

Aside from one farmer who resented that he had to vaccinate against rotavirus after buying an infected calf (F10, farm manager), most conversation about vaccination was focused on pneumonia. Vaccinating calves against pneumonia was believed to help mitigate the impact of subpar calf accommodation, but a veterinarian was frustrated by the reluctance of farmers to vaccinate against pneumonia despite rearing calves in poor environmental conditions.

“There’s a couple of people who have horrible pneumonia problems and the shed’s not designed for [calves]; they’re overstocked and they’ve mixed different age groups—so they’re doing everything wrong and yet they still won’t vaccinate and it’s just madness!”

V8, farm veterinarian.

Economic considerations contributed to the absence of calf vaccination on some farms.

“We haven’t recovered properly from the [milk] price slump yet, and so to start a new vaccination policy and everything like that, I just don’t think it would be looked upon well.”

F22, herd manager.

Several participants were vaccinating calves against pneumonia (Table S1) but the perceived effectiveness of pneumonia vaccines was mixed.

“Some years, we were treating virtually all the youngstock [for pneumonia], whereas now we get one, two. So [the vaccine has] made a great difference.”

F15, calf rearer and farm worker.

“It’s very frustrating because we’ve started vaccinating everything for pneumonia but yet we still have to treat a lot of [calves] with pneumonia.”

F24, farm manager.

One farmer (F9) took blood samples to assess the effectiveness of their pneumonia vaccination programme.

“We regularly take blood samples of calves that contract pneumonia, even though they’ve been vaccinated, to try to identify the strains and make sure that it’s being covered by the vaccine, or if it wasn’t administered correctly, or if the vaccine simply didn’t work.”

F9, farm manager.

One veterinarian questioned whether pneumonia vaccines would be necessary if calf facilities and early life management were adequate.

“We’ve got any number of vaccines available, and yet they still don’t cover all the infectious causes of pneumonia. And we keep getting hammered by drug companies that we don’t sell enough pneumonia vaccine! Well actually, if we got the colostrum right, and we got the feeding right, and we got the environment right, we wouldn’t need any!”

V8, farm veterinarian.

However, despite a keen focus on colostrum management and recent investment in a new youngstock unit designed with the help of their veterinarian to promote good calf health, this farmer still had to treat calves for cases of pneumonia.

“Part of the expectation of the new calf unit was that we would not need to vaccinate any more … We did have an element of pneumonia in the new calf unit last year, which was disappointing.”

F26, farm manager.

### 3.2. Management of Calf Environment

#### 3.2.1. Calf Group Management

Farmer participants housed calves in a variety of group sizes in outdoor hutches, indoor pens, or a combination (Table S3), with a slight majority of the interviewees using group housing of calves, in a mixture of pens and hutches. This variation was largely dependent on the space available to rear calves and the labour-intensiveness of different systems. Individual hutches were considered particularly demanding, but worth the extra labour for improved calf health:

“I’m highly satisfied with all disease levels. Diseases are pretty low in their hutches.”

F7, calf rearer and farm manager.

Calves were often individually housed at first, and then grouped once they were confidently drinking milk or after weaning (Table S3), but the way in which calves were grouped was also somewhat dependent on the priorities and time management of the responsible stockperson(s).

“I wanted to make sure everything went through a single hutch and then went on to group hutches … you end up cleaning out a group hutch, put some calves in, then you have to clean out [the single hutches] as well. Before I came, they didn’t really bother doing that. They just cleaned out the group hutch and then just put the new [calves] in there instead.”

F3, calf rearer and farm worker.

While the social interaction of group housing could be beneficial for calves, it was also thought to result in some being bullied away from feeding by other calves, which led to variable growth rates within the same group.

“I do like putting them into the big hutches and seeing them run around and seeing them mix and interact, but it is just interesting that we’ve had real variance in growth rates from the group hutches [prior to weaning compared to individually housed calves in a trial].”

F1, calf rearer.

Grouping calves was also perceived to influence disease incidence, although it was suggested that disease transmission could be mitigated by appropriate management.

“A lot of the guys will pen individually and feed individually and I think that massively reduces scours, but then probably increases pneumonia further along.”

V4, farm veterinarian.

“It’s important to keep them in very small groups of a similar age, and don’t mix them.”

F10, farm manager.

“Instead of trying to limit it to number, limit it to age range. If we think of how the diseases spread, it’s normally a 10 day to 2 week cycle. So if you open a pen and only fill it for 2 weeks and then shut that pen, independent of whether it’s got three calves in it or 10 calves, which is really the maximum I like to go with, then that’ll stop your disease spread.”

V11, youngstock veterinarian.

Some farmers noted the apparent ease of disease transmission between calves and from older animals, and so were mindful of internal farm biosecurity. Several farms kept heifer calves and beef calves separate to avoid cross-contamination, and a few considered the equipment being used for both the milking herd and the youngstock:

“One thing that we are quite careful with is that we use the loader that hasn’t been in all the muck where all the Johne’s is. A lot of farms, they’ll do everything for Johne’s, but then they’ll just drive in and scrape up with the tractor that scraped up all the cow muck.”

F1, calf rearer.

“We do go from adult cows to young calves. We don’t go in the pens with the calves without washing our wellies [boots].”

F9, farm manager.

However, several farmers struggled to maintain sufficient space for all their calves:

“Overstocking is probably the biggest issue … we had so many I had to double up all the single hutches.”

F3, calf rearer and farm worker.

This was sometimes due to farms being under movement restriction due to bTB, resulting in limited outlets for the sale of their calves and high stocking densities on farm. This often compromised hygiene and grouping/housing protocols, and, in some cases, it resulted in calves sharing airspaces with older cattle.

“One of the sheds that we were moving calves into had older cattle in, because we were a bit tight for space. The vet said that ’older cattle are resistant to a lot of the pneumonias, but of course they’re still breathing out the virus, so then if you’re putting youngstock in with older ones, then you’re at high risk of passing on.’ So we’ve tried to break that link [by putting up some new sheds as calf housing].”

F5, farm manager.

#### 3.2.2. Thermal Comfort and Hygiene within Calf Accommodation

Several farmers aimed to create a microclimate for calves to provide them with thermal comfort. Some farmers considered modifications within the accommodation itself to help keep calves warm, but actions were influenced by the perceived severity of seasonal weather.

“We did think about putting a cover over the back, like they do with pigs, with some straw on top to keep them warm. The vet suggested it, actually. We might do it, but it hasn’t been too bad this year; we’ve had a reasonably kind winter.”

F20, farm manager.

Standard use of calf jackets was more common during the winter in all-year-round calving systems rather than block calving systems (Table S3), due to the number of calves requiring jackets at the same time in block systems. Some farmers kept a small number of calf jackets to aid the recovery of ill calves.

“We’ll put a calf jacket on anything that has had the scours, really, and just looks generally not very thrifty.”

F20, farm manager.

However, use of calf jackets was dependent upon the stockperson’s knowledge of how to make best use of them. One farmer (F9, farm manager) used two different brands of calf jackets, noting that the sizes available from one brand were too large to benefit small calves. Another farmer was unsure of the calf jacket protocol to follow if they were not standard attire for all calves.

“I don’t know when you would take a calf jacket off. Some people say once it goes on, it stays on until you’ve weaned them … But if you put a jacket on like for a week, and then take it off, does a calf go back [lose condition] then? I don’t know.”

F14, calf manager.

Although bedding plays a key role in thermal comfort for calves, farmers seemed to focus on the aesthetic and disease prevention associated with providing calves with plenty of clean, dry bedding.

“I don’t want the calves to ever look dirty … If they look dirty, I’m a day late with the straw going in … I get moaned at by dad because he thinks I use too much straw, but it saves me [using] antibiotics.”

F19, farm manager.

Adding fresh bedding on top of soiled material was acknowledged by some to “breed more bugs” (F14, male calf rearer), but was a common practice. Stockpersons might postpone cleaning the calf pens, especially where calf buildings did not allow for easy hygiene management.

“I’ve had a lot of coccidiosis in that one particular pen, but you can’t just go and clean that one pen out … you have to clean the whole shed out. Well, then you sort of think ‘it can wait another week’. Things like that don’t get done as quick as what they should because it’s quite a job to push everything out, take all the pens down, then clean out, then put it all back up again.”

F14, calf rearer.

Participants noted that pathogen load could be further reduced if calf housing could be disinfected and left empty for a time, but this option was limited by the space available to house calves on farms.

“We don’t have enough space, so we can’t have [the pens] resting. It’s a day’s rest. They’re cleaned out, they’re sprayed with peracetic acid, washed down with it, and then left ‘til they dry, but it’s not that long. A nice drying day like today helps. A wet, drizzly day and they don’t really ever dry out.”

F19, farm manager.

In block calving systems, it was possible to rest calf accommodation between calving blocks, although disease burden did build up over the course of the calving season.

“Leaving a shed clean, dry and empty for a few months massively reduces the pathogen challenge … You see a lot less disease, especially at the start of the block. It might build up towards the end of the block, but compared to these guys who are constantly housed, it definitely helps.”

V3, youngstock veterinarian.

#### 3.2.3. Investing in Calf Accommodation

Many farms had limited space for calf rearing and often utilised existing multipurpose farm-buildings to house calves with inappropriate airflow and drainage, partly because the milking herd and parlour were commonly prioritised for investment.

“Access to clean out the shed is very difficult … And the floors, they should be on more of an angle … but they’re reasonably flat, so drainage into all the drains isn’t particularly great. It’s not the best calf shed, really, considering we’ve got this nice dairy.”

F24, herd manager.

“Most buildings in the UK are old buildings that you use for calves. You’ll spend money on your buildings for your cows, but you won’t spend it on the calves. Calves go in some poorly ventilated, or cold, damp area.”

V2, youngstock veterinarian.

Often, the person working with the calves was not in control of the farm’s finances, so they had little choice but to work within the limitations of the calf facilities available to them. Farm developments were in competition for space, function, and expense; hence, even if calf rearers were consulted, their input was restricted to a choice between what they perceive to be suboptimal options for calf accommodation.

“It all comes down to money at the end of the day; so, it’s a shed here empty, so we use it and you’ve gotta make the most of it and just get on with it … We’ve got a couple of those Igloo things [group hutches], I hate them … I’ve never seen so many ill calves … [The farm manager] said we could put a concrete slab there and use those Igloos and I said, ‘Nah—I’d rather use this [shed that’s not ideal]!’”

F23, farm worker and calf rearer.

In many cases farmers appreciated an advisor’s ability to recommend practical, realistic upgrades to existing accommodation to improve calf health.

“Some vets have this similar sort of mindset: in an ideal world you could do [with] a new space, well it’s not an ideal world, so what are we gonna do? Some don’t have that, they come out with theory … we all read the same books, but how do we get different results [on our farm]?”

F25, farm manager.

All participant farmers who had invested in purpose-built calf accommodation perceived significant improvements in calf health. However, in many cases, erection of new calf housing was considered prohibitively expensive. The decision to invest in new calf housing was largely dependent on the farm’s willingness, or ability, to finance the development.

“Eventually we came to the conclusion that we had to spend some money, this [the new calf accommodation] was desperately needed [to improve calf health].”

F26, farm manager.

“The farmer may know that the shed he rears the calves in is just awful … but he also knows he hasn’t got *x* thousand pounds to put up a new one … He’d need to be very convinced that if he goes out and borrows *x* amount to put up a calf house that there is gonna be a return that will pay for his borrowings, and that can be a challenge.”

V10, formerly practising veterinarian, now feed consultant.

Even where the farmer and veterinarian were discussing improvements to calf accommodation, financial constraints could halt progress. The same suboptimal accommodation would be used, calves would continue to require pharmaceutical treatments, and stagnation contributed to despondency, for both the farmer and the veterinarian, at not being able to progress with preventive calf health measures.

“Sometimes you turn up to what feel like slightly helpless situations, where they’re going, ‘I know this shed is awful … I can’t deal with it now [because of financial pressures].’ … It can reach a brick wall where people are much happier to go, ‘Well it’s broken, we’ll just use the drugs’, than to really start investing their time and energy in patching together that shed.”

V6, youngstock veterinarian.

#### 3.2.4. Designing Replacement Calf Accommodation

Advisors stressed the importance of building accommodation with a focus on calf health, recommending veterinary involvement at the designing stage of the development, which many farmers had not done.

“I have seen plenty of big, shiny units … that don’t necessarily perform as well as they were hoped by the person who designed them … People get advice from different sources, and often the animal health side of things only actually comes in once you’ve got animals in the shed and maybe they’re not performing.”

DR1, pharmaceutical company veterinary advisor.

“If they could involve the vet more in building planning … I think we could save them thousands and thousands of pounds, but it’s often one of the last people that a farmer will consider speaking to is their vet when they’re putting up a new shed … Shed design is probably not something they think that we know an awful lot about.”

V4, farm veterinarian.

Relying on building contractors was reported to be problematic since they are unlikely to be familiar with the scientific basis for the design features of a calf shed. It was considered important that the farmer, potentially with support from their veterinarian, was confident in the rationale behind building design elements to ensure the accommodation was built according to specifications likely to optimise calf health.

“[A farm client] building this new shed … that had a 1 in 20 slope … When they were building it, he called me out because the builders were going, ‘We will do it, not a problem, but [1 in 20 is a very steep incline] on your head—are you sure?’ … If we commit the cardinal sin that has led to these sheds in the past of looking at it and going, ‘Phwar, bit steep, maybe a little less?’ then … it’ll still be 30,000 GBP [42,323 USD], it just won’t work as well as it might.”

V6, youngstock veterinarian.

However, calf health was not always a priority for farmers when building calf accommodation. One dairy bull calf rearing enterprise (F17) prioritised having buildings which were multipurpose to allow adaptability in function in response to volatile market fluctuations.

“The whole sheds are designed with multipurpose in mind. As time’s gone on, they’ve become more angled towards calves, but if things changed tomorrow and the money dropped out of calves, it’d probably take us a week to convert this shed into a pig shed.”

F17, farm worker on calf rearing unit.

Another farmer used their own initiative to design and build affordable calf accommodation, replacing their previous set-up of 12 calf hutches which did not allow them enough calf rearing capacity. His innovative design was popular with other farmers, likely due to his focus on cost-effectiveness and ease of management.

“Cost 7000 GBP [9876 USD] to build; that’s everything, metalwork, concrete panels. We can fit 42 calves in here … A lot of farmers would need to [get input from the veterinarian]. I went online and looked it up; it’s all on the internet … We wanted to make physical barriers so then we could … take this pen out, steam clean it, and that pen can still be there! … I know of two farmers that have copied it since we’ve done it.”

F20, farm manager.

### 3.3. The Role of Stockmanship and Perceived Control

Attention to detail in calf rearing was stated by every participant to be the most important aspect for successful calf rearing, particularly with regard to disease management, and it was dependent on the skill, time, and interests of the stockperson, as well as the facilities available to them.

“It’s just little things that make such a big difference to calf rearing … if you’ve got a problem, deal with it straight away, and if you can move them to a fresh place, a fresh, clean place, that makes a huge difference.”

F2, calf rearer.

Stockmanship was commonly perceived to determine how well calves could perform in any building.

“I’ve walked into some sheds that I have thought ‘[swears], this is an awful place to see calves’, and actually, when you look at the calves, they are growing really well—you can’t put a value on good husbandry.”

DR1, pharmaceutical company veterinary advisor.

“You could have the most amazing shed in the world, but if you don’t have attention to detail of like the stupid little things … you’re never gonna get it right.”

F18, calf rearer.

**Figure 1:**
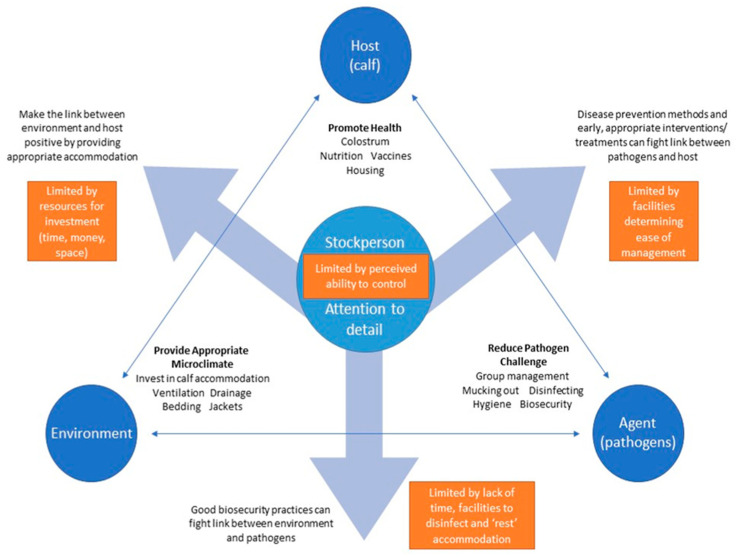
Schematic demonstrating the human dimension of the epidemiological triad.

This emphasises the importance of human influences on calf husbandry and health in the context of the epidemiological triad of interactions among the host, pathogen, and environment, as modelled in Figure 1.

#### Perceived Control over Disease Processes

Farmer participants endeavoured to prevent calf disease from occurring on their farms. Once disease issues are established, it becomes a difficult cycle to escape.

“If you’ve got unhealthy calves, it doesn’t matter what you do, you’re on a backwards spiral all the time. They’re not very well, then they don’t drink [milk], so then they don’t gain weight, and because they’re not getting that adequate nutrition, you get more health issues.”

F2, calf rearer.

Despite investing in preventive measures, health problems—especially pneumonia—often persisted on farms. This could leave farmers disillusioned and wondering what more they could do to address the issue.

“We get pneumonia every single month of the year—even in the middle of summer … We vaccinate for it [pneumonia], we’re looking out for it all the time, we never lose any with it, but we do jab [inject] a lot of calves for pneumonia. There’s no sort of pattern to it … they’re bedded up well, but we still get it.”

F8, farm manager of dairy bull calf rearing unit.

Furthermore, weather conditions were thought to contribute to pneumonia because “it doesn’t matter how good your ventilation is … you’re still pumping cold, damp air into a building” (F13, farm manager), and difficulties in determining what specific aspects of calf management needed attention to improve the situation also appeared to contribute to a perceived lack of control over disease incidence.

“Dad had two [calves] the other day that didn’t do very well. I don’t know what happened there; they looked like calves that missed their colostrum.”

F19, farm manager.

One farmer implied that experiencing a small number of calf deaths was inevitable.

“It’s rare that you get one die, I mean, you always get the odd one”

(F14, calf rearer).

However, another farmer believed that “mortality’s usually a result of bad management” (F20, farm manager). This perception might partially relate to the disease profiles of individual farms; F20 was accredited as BVD-free, whereas F14 had a low level of BVD within their herd. The immunosuppression caused by BVD could make it difficult to successfully rear calves.

“I think it’s a waste of time rearing heifer calves if you have got BVD … We don’t have BVD so we’ve not got that sort of threat on them being pushed towards them getting pneumonia and scours and all that business.”

F10, farm manager.

“You see some farms where they keep their calves in appalling conditions and never have any problems because … there’s no BVD.”

V8, veterinarian.

## 4. Discussion

The research findings presented in this paper once again highlight the essential human dimension of disease management in calves [58]. Interviewees alluded to interactions among each of the three components of the epidemiological triad (host, pathogen, and environment) [27] in relation to calfhood diseases, but these interactions are influenced and controlled by human actions or interventions. Stockmanship was believed to help mitigate the effects of suboptimal calf accommodation; excellent facilities could support—but not replace—good calf husbandry and attention to detail in calf rearing. The interest and aptitude of the stockperson for calf care enabled them to notice and deal with problems promptly, preventing them from developing into permanent crises [41], and this fostered enjoyment of the work, valued as important by dairy farmers [38]. However, causal factors for disease were often difficult to pinpoint; thus, it could be challenging to decide which specific curative actions to take, especially where calves were immunocompromised due to BVD [14].

In the present study, the efficacy of calf rearing was further challenged by limited resources including time and finance. This could contribute to a perceived lack of control and inability to improve the health of calves, resulting in inaction [40]. Whilst it is important for research to consider the practices and facilities which can promote good calf health—and there has been much research in this area [4,59,60]—the individuals responsible for providing calf care must not be overlooked [45,61,62]. Farmer-led participatory approaches, where farmers are facilitated to learn best practice from peers, can motivate and empower them to make changes and regain control [63,64], suggesting that these approaches could be beneficial in achieving the continuous improvement of rearing practices resulting in better calf health and welfare.

Farmer participants in the present study tended to perceive low levels of calf mortality on their farms. Calf mortality on UK farms was reported previously as low as 4.5% [7] and as high as 48% [6], which could suggest that participants in this study may have downplayed or underestimated their calf mortality rates. Santman-Berends et al. [40] found that, on farms experiencing high calf mortality, farmers were often unaware of the issue. Nonetheless, research has also shown a range in mortality rates of 0–30% across farms, suggesting that good husbandry can mitigate the effects of disease [4]. Due to the non-random nature of sampling for this study and willingness to be interviewed on the topic, participants may have had a keen interest and focus on calf rearing; it is, therefore, possible that participating farmers actually achieved the low levels of calf mortality which they perceived. Mortality rates may also be affected by farmers’ pre-emptive actions; some believed that it was more cost-effective to euthanise calves experiencing ongoing illness. Culling may not be perceived as mortality per se, rather serving an economic purpose [65]; however, whether calves die directly from disease or are euthanised because of disease, the end result is the same. The perceived cost–benefit of treating versus culling calves may also be linked to the stockperson’s ability to identify initial signs of disease and administer early treatment, a key contributor to preventing treatment failure [22,66], recurrence of illness, and long-term damage [66]. Early disease detection and intervention could be aided by technology, and there is potential for further use of technological management aids for dairy calves, in line with precision farming approaches being developed for disease detection in adult cattle [67,68].

In agreement with the existing literature (reviewed by Johnson et al. 2011 [4]), calfhood pneumonia and diarrhoea were considered the most problematic and/or common calf health issues encountered in the present study. Farmer participants tended to perceive pneumonia to be the most problematic, but advisors indicated that scour was a key problem which was often underestimated by farmers. Participating farms may well have experienced higher incidences of pneumonia compared to enteritis, but it has been previously noted that diagnoses by farmers are often inaccurate and underestimated [4,59] and records might lack detail [66], affecting farmers’ perceptions of the main problems on their farm [59,69]. Whereas UK farmers are legally obligated to record pharmaceutical treatments of livestock [70], including antibiotics and NSAIDs (the main treatments associated with BRD [23]), there is no mandate to monitor the use of oral rehydration therapy (the primary treatment for calf scour [18,19]). Furthermore, some farmers participating in the present study were aware that calfhood pneumonia could negatively impact animals’ long-term health, welfare, and longevity [2], whereas the long-term effects of diarrhoea in calfhood, which have been shown in previous research [71], were not mentioned. This lack of appreciation of the long-term impact of gastrointestinal disease may contribute to the perceived greater importance of respiratory disease alluded to by interviewees. In addition, farmers might perceive scour as less problematic because they consider it comparatively easy to control through improved hygiene management [18], whereas pneumonia prevention was considered to require investment in building infrastructure and was more affected by the weather and climatic conditions [23].

In the present study, more of the farmers interviewed vaccinated calves against pneumonia compared to enteritis, but several questioned the efficacy of pneumonia vaccines; similar findings were reported in a recent UK survey about youngstock rearing and disease [6]. Vaccine efficacy might be impinged by improper storage [72] or administration [73], but the complex nature of BRD and its environmental interactions leaves the (cost-) effectiveness of vaccination arguably uncertain [23]. The causal pathogens for pneumonia are more difficult to diagnose [4]; therefore, farmers more easily and frequently referred to the potential causes of diarrhoea. Usually relying on historic diagnoses on the farm rather than testing faecal samples from every future scouring calf, the farmers were seemingly concerned that the time taken to obtain results would delay treatment. On-farm diagnostics such as the Rainbow^™^ Calf Scour test (Bio-X Diagnostics S.A., Rochefort, Belgium) can detect four of the main causal pathogens (rotavirus, coronavirus, *E. coli* F5 (K99), and *Cryptosporidium parvum*) in calf stool within 10 min and could be incorporated into standard treatment protocols to ensure appropriate treatments are given. However, some participating farmers, despite attributing the diarrhoea to cryptosporidiosis or coccidiosis, reported treating cases of scour with antibiotics; Baxter-Smith and Simpson (2020) [6] found that 27% of surveyed farmers used antibiotics to treat diarrhoea. Routine treatment of calf diarrhoea with antibiotics has been shown to have minimal or negative effects and, hence, is not recommended [4] unless calves are systemically ill [18,74]. However, antibiotics were previously recommended as standard treatment and used indiscriminately [75], and challenging these established, habitual practices is difficult [63]. Improving protocols around vaccinations and antibiotic treatments in calves is an essential part of antimicrobial stewardship, but it is necessary to consider farmer opinions and mindset, as well as technical issues [61], and the approach of the veterinarian can influence behaviour change in farmers [76].

Participants identified calf housing as a key influencing factor for calf health, as has been noted elsewhere [30]. A European study published in 2010 [77] reported that 60% of UK herds used individual pens for calves, but anecdotal evidence suggests that this trend is moving towards paired and group housing, as supported by the findings in our study, where individual housing was used in a slight minority of the participating farms. Individually housing calves, especially in outdoor hutches, was considered by some to be beneficial for calf health, particularly in the first days–weeks of life, but these systems are more labour-intensive compared to group housing [78], and health risks associated with group housing calves can be mitigated by appropriate management alongside good stockmanship [31]. However, many farms used pre-existing, multipurpose buildings to accommodate calves, requiring stockpersons to manage within an environment with inadequate airflow and drainage, which can predispose calves to contracting pneumonia [79]. Poor building design, lack of space, and all-year-round calving affected the ease and, therefore, frequency of conducting basic hygiene practices such as cleaning out and disinfecting pens, and they prevented the implementation of an all-in/all-out system [80], exposing calves to greater risk of disease.

Similar to previous findings [6], farmers in this study often identified housing, stocking density, facilities, and ventilation as areas for desired improvement. Since design features can allow for easier management within an optimal calf environment to foster good calf health [30], participants who had installed purpose-built calf accommodation perceived it to be a worthwhile investment. However, in many cases, replacement accommodation was highlighted as necessary but prohibitively expensive or not cost-effective [81]; thus, farmers continued “making do” with suboptimal facilities, sometimes making alterations to improve existing calf buildings, usually to improve ventilation. These relatively minor changes were generally considered easier and less costly to implement, but they were also less effective than a complete overhaul of calf accommodation. Thus, lack of funds preventing structural improvements could lead to disillusionment [40], potentially resulting in an over-reliance on antimicrobials to make up for farm deficiencies. If consulted, veterinarians were often expected to offer practical, realistic recommendations that were possible to achieve within farm constraints of space, labour, and financial considerations, but farmers indicated that some veterinarians were more able to put theory into practice than others. Veterinarians were concerned that they were not often consulted about building design, and previous findings have indicated that farmers do not perceive veterinarians as important consultants on this topic [82]. Large financial investments in purpose-built calf accommodation may, therefore, not be as effective as they could be in promoting good health in calves. It is also possible that the cost of replacing suboptimal calf accommodation need not be as great as some participants perceived; one farmer was able to research, design, and build affordable calf accommodation with a focus on functionality, suggesting that, by clever sourcing of materials, lower-cost housing solutions may be possible in the mainstream. Facilitated farmer-led approaches harness the interests and motivations of farming peers to help others; they have proven effective in developing practical innovations relating to a range of topics [64,83] and could potentially be used to create more cost-effective building solutions for calf housing.

The essential role of good stockmanship and attention to detail in maintaining calves in good health (as represented in Figure 1) must not be underestimated. Research surveys tend to focus on the prevalence of calf management practices relative to an area of interest, e.g., use of automated milk feeders [84], or their associations with mortality and morbidity [59,85]; however, the diligence with which stockpersons carry out these activities, i.e., the level of attention to detail, remains unclear. Furthermore, the concept of “attention to detail” is applied broadly across all areas of farm performance, planning, and day-to-day management [86] and is not well defined. From the farmers’ perspectives, attention to detail appears to mean doing the small things well [87], such as noticing and responding to early signs of illness, and maintaining good hygiene practices [44]. Others have defined “attention to detail” as knowing the value of specific activities and managing time accordingly, resulting in the aggregation of marginal gains [88]. It is recommended that goals should be SMART (specific, measurable, actionable, relevant, and time-bound) [89]; therefore, the concept of attention to detail should be applied to a specific context or activity. To the authors’ knowledge, the concept of “attention to detail” as it relates to animal management has not been explored in depth; it remains a vague term, despite its apparent importance. It is likely that what constitutes “attention to detail” is interpreted differently according to individual interests and the requirements of different roles. For example, some, such as calf rearers, might prioritise calf-based observations which allow for immediate, specific actions as part of day-to-day management [43,44]; others, such as advisors and farm managers, might seek details which offer broader, long-term insights, for example, to aid farm health planning or business decisions [52,86]. To navigate these different priorities relating to calf rearing and, more specifically, disease management in calves, facilitation could be a useful tool as it can help actors to navigate difficult, multifactorial issues [63], and investing in trained facilitators can aid decision making and guide farm actors through a process of change leading to continuous improvement [63,90].

## 5. Conclusions

Calf pneumonia and diarrhoea were the main problems experienced by participants, but it was believed that the severity of calf health issues could be minimised by paying close attention to detail with respect to the husbandry of calves and management of their environment. On some farms, suboptimal calf facilities and reluctance to invest in protective measures impeded actions to protect calf health and could limit the success of attempted mitigation strategies, leaving stockpersons and advisors feeling helpless to change the situation. More efforts need to be directed to promoting health and immunity in calves, improving the microclimate around them, and reducing pathogen challenge in their environment. Achieving improved calf health and welfare on farms is dependent upon fostering perceived control and self-efficacy in farmers and stockpersons. This could be achieved by using supportive knowledge exchange practices including farmer-led participatory approaches and facilitation. Further research is needed to better understand what “attention to detail” means to different actors within specific farm contexts. Although the findings are based on dairy farms in England, they are likely to be indicative of opinions and experiences elsewhere in the world. It is essential that efforts to promote disease management practices not only focus on technical solutions, but also seek to positively influence the mindset, priorities, and experiences of the persons responsible for calf rearing, as well as the allocation of farm finances, if dairy calf health and welfare is to be improved at a national and international level.

## Data Availability

The data are not publicly available as participants did not consent to this as part of their informed participation in the study, and the anonymity of participants may be put at risk by releasing interview data in full.

## Supplementary Materials

The following are available online at https://www.mdpi.com/article/10.3390/ani11102829/s1: Table S1. Participants’ main health problems in their calves and prevention methods; Table S2. Participants’ main treatment protocols; Table S3. Participants’ calf housing, group management, and hygiene practices.
