# Supplementary material for: Stakeholder Perceptions of Disease Management for Dairy Calves: “It’s Just Little Things That Make Such a Big Difference”
Source: Animals (Basel). 2021 Sep 28;11(10):2829. doi: 10.3390/ani11102829 (PMC8532611; doi:10.3390/ani11102829)
Supplement: Supplementary file 1 [file animals-11-02829-s001.zip › animals-1389017-supplementary.pdf]

Table S1 Participants' main health problems in their calves, and prevention methods.

Farm	Problems, past and present	Main problem(s)	Vaccines	Other prevention methods
**F1**	Cryptosporidiosis, scour, pneumonia (adults with lung damage)	No major issues	Against pneumonia, product not specified.	- Halocur® [1] oral treatment first three days<br>- Treat navel<br>- Attention to detail
**F2**	*Mycoplasma*, pneumonia, scour	No major issues	Against pneumonia:<br>- Bovilis® Ringvac [1]<br>- Rispoval® RS+PI3 Intranasal [2] (stopped last year - no issues)	- BVD tag and test<br>- Treat navel<br>- Attention to detail<br>- Cleanliness
**F3**	Pneumonia, scour	Pneumonia (overstocking)	Against pneumonia:<br>- Bovilis® Bovipast RSP [1]<br>- Intranasal (not specified)<br>Against scour:<br>- Bovilis® Rotavac® Corona [1] (cows at drying off)	- Treat navel
**F4**	Cryptosporidiosis, Coccidiosis, pneumonia	Scour	Against BVD and leptospirosis, products not specified.	- Treat navel<br>- Cleanliness
**F5**	Pneumonia, pot bellied calves	Pneumonia	Against pneumonia:<br>- Currently Rispoval® 4 but planning to change to RS+PI3 Intranasal [2] and vaccinate cattle against BVD<br>- Against IBR, product not specified.	- Once vaccine protocol changes, will do BVD tag and test
**F6**	Coccidiosis	No major issues	Against pneumonia/BVD:	- Treat navel

			- Rispoval® 4 [2]<br>Against lungworm:<br>- Bovilis® Huskvac [1]<br>Against black leg and leptospirosis. Products not specified.	- Attention to detail<br>- Clean boots before entering calf space
**F7**	Navel-ill, Joint-ill, respiratory disease	No major issues	Against pneumonia/BVD:<br>- Rispoval® 4 [2]	- BVDFree accredited<br>- Monitoring faecal samples for Coccidiosis<br>- Treat navel
**F8**	Pneumonia, Cryptosporidiosis, scour	Pneumonia	Against pneumonia:<br>- Rispoval® RS+PI3 Intranasal [2]	- Calves treated with 4 ml Selectan® [3] (Florfenicol-based injectable antimicrobial against pathogens causing BRD) on arrival
**F9**	Pneumonia, Cryptosporidiosis, Coccidiosis	Pneumonia	Against pneumonia:<br>- Rispoval® RS+PI3 Intranasal [2]	- BVD tag and test (heifers only, bull calf buyer sees no merit in it)<br>- Good hygiene<br>- Fans for increased ventilation
**F10**	Rotavirus	No major issues	Against scour:<br>- Rotavirus, product not specified.	- Free of BVD
**F11**	Pneumonia, scour	No major issues	Not covered.	- Free of BVD (but considering stopping tag and test due to cost).<br>- Attention to detail
**F12**	Pneumonia, Rotavirus, Salmonella	No major issues	Against scour:<br>- Rotavirus, product not specified.	- Cleanliness<br>- Treat navel
**F13**	Pneumonia, scour	Pneumonia	Against pneumonia:	- Keep bedding clean and dry

			- IBR<br>- BVD<br>- Against leptospirosis Products not specified.	- Improved ventilation<br>- Outdoor rearing<br>- Starting BVD tag and test
**F14**	Pneumonia, *Mycoplasma*, Infectious Bovine Rhinotracheitis (IBR), coccidiosis, leptospirosis,	Pneumonia	Against pneumonia:<br>- Rispoval® RS+PI3 Intranasal [2]<br>- Against IBR, product not specified.<br>Against lungworm:<br>- Bovilis® Huskvac [1]	- Low levels of BVD according to bulk milk tests, will start blood testing heifers as part of BVDFree England scheme.<br>- Fans for improved ventilation<br>- Vecoxan® [4] (Diclazuril-based endoparasiticide oral drench against coccidiosis) in final milk feed prior to weaning
**F15**	Pneumonia, Coccidiosis	Pneumonia	Against pneumonia:<br>- Rispoval® RS+PI3 Intranasal [2]	- Low levels of BVD according to bulk milk tests, "testing really poorly calves [for] BVD, and we always keep coming negative on that"
**F16**	Minor nutritional scour	No major issues	Not covered.	Not covered.
**F17**	Pneumonia, coccidiosis	Pneumonia	Against pneumonia:<br>- Bovilis® Bovipast RSP [1]	- Deccox® [2] (Decoquinate-based endoparasitiide against cocidiosis) at a low level in concentrate for 4 weeks upon arrival.
**F18**	Pneumonia, rotavirus, cryptosporidiosis, coccidiosis, salmonella	No major issues	Not covered.	- BVD tag and test, no positive results so far<br>- Good hygiene<br>- Attention to detail
**F19**	Pneumonia, cryptosporidiosis, nutritional scour	No major issues	Not covered.	- Free of BVD<br>- Vecoxan® [4] in milk when case(s) of coccidiosis occur.
**F20**	Pneumonia, cryptosporidiosis	Pneumonia	None. May start vaccinating to reduce antibiotic treatments for	- Free of BVD<br>-"more airy sheds"

			pneumonia	
**F21**	Pneumonia, coccidiosis, rotavirus	No major issues	Against pneumonia:<br>- (unspecified Rispoval® [2])<br>Against scour:<br>- Rotavirus<br>- Against ringworm Products not specified.	- BVD tag and test, no positive results for past 3 years<br>- Powdered antibiotic (product not specified. To help reduce scour related to cryptosporidia and coccidiosis)<br>- Hygiene<br>- Improved calf housing
**F22**	Pneumonia, general ill-thrift, nutritional scour	Pneumonia	Against BVD and leptospirosis, products not specified.<br>Stopped pneumonia vaccine due to price pressures.	Not covered.
**F23**	Pneumonia, swollen navel, diptheria	No major issues	Against pneumonia, product(s) not specified.	- Fans for improved ventilation
**F24**	Pneumonia, scours	Pneumonia	Against pneumonia, product(s) not specified.	- Improved ventilation
**F25**	Scour, pneumonia	No major issues	Not covered.	- Halocur® [1] oral treatment in first milk feed.<br>- Attention to detail
**F26**	Pneumonia, Coccidiosis, Cryptosporidiosis, Rotavirus	No major issues, pneumonia	None. Stopped pneumonia vaccine due to new calf housing.	- Good hygiene<br>- New, purpose-built calf housing<br>- Attention to detail

Note: The contents of this tables are not exhaustive, other practices may not have been mentioned in the conversation.

[1] MSD Animal Health UK Ltd.

[2] Zoetis UK Ltd.

[3] HIPRA UK & Ireland Ltd.

[4] Elanco UK Animal Health Ltd.

[5] Norbrook Laboratories Ltd.

[6] Boehringer Ingelheim Animal Health UK Ltd.

Table S2 Participants' main treatment protocols.

**Warning sign/Illness**	**Treatment protocols**	**Farms citing these treatments**
Slow drinking calves	- Thermometer: check for fever then select appropriate treatment.	F1, F19, F24
Calves 'not quite right' (behaviour, early symptoms)	- Thermometer: check for fever then select appropriate treatment.	F2
Pyrexia	- Treat with Metacam® [6] and oxytetracycline antibiotics (product not specified).	F24
Calf Scour	- Separate scouring calf from group<br>- Add oral rehydration sachet to milk fed little and often<br>- Oral rehydration (route of administration not specified)<br>- Oral rehydration therapy and antibiotics (product not specified).<br>- Treat scour with electrolytes and in cases of coccidiosis, provide Norodine® [5] (antibiotic).<br>- Treat cases of cryptosporidiosis with Halocur® [1]	F6, F25<br>F6, F25<br>F26<br>F23<br>F25<br>F19<br>F20
(Early) calf pneumonia symptoms (breathing, coughing, high temperature)	- Treat with antibiotics (product not specified).<br>- Oral rehydration therapy and antibiotics (product not specified).<br>- Inject with long acting antibiotic and anti-inflammatory.<br>- Metacam® [6] anti-inflammatory and antibiotics if necessary (product not specified, treatment protocols based upon advice from veterinarian).<br>- Alamycin®[5] (long acting antibiotic containing Oxytetracycline Dihydrate) or Draxxin® [2] (Tulathromycin-based antibiotic) and Metacam® [6] (Mexoxicam-based NSAID)<br>- Resflor® [1] (Contains Florfeniol and Flunixin to provide antibiotic, anti-inflammatory and anti-pyrexic in one dose)<br>- Treat cases of pneumonia with Zactran® [6] (Gamithromycin-based antibiotic) and pain relief from Metacam® [6]<br>- Treat cases of pneumonia with Metacam® [6] to reduce pain and Nuflor® [1] (Florfenicol-based	F20<br>F23<br>F2<br>F26<br>F11, F22<br>F7<br>F13<br>F17

	antibiotic) to treat the infection.<br>- First case give 5ml Resflor® [1], repeat case give Draxxin® [2] and Metacam® [6], if treatment fails again, Alamycin® [5]	F25

Note: The contents of this tables are not exhaustive, other practices may not have been mentioned in the conversation.

[1] MSD Animal Health UK Ltd.
[2] Zoetis UK Ltd.
[5] Norbrook Laboratories Ltd.
[6] Boehringer Ingelheim Animal Health UK Ltd.

Table S3 Participants' calf housing, group management and hygiene practices.

Farm	Farm details: Calving Pattern, Herd Size, Farm System	Accommodation	Group size(s)	Cleaning practices	Jackets worn by calves
**F1**	AYR, 380, conventional	Hutches	Individual until after weaning, then groups of 5	Fresh bedding every 3 days, pressure washed, steam cleaned and disinfected (using Kilcox® disinfectant - effective against coccidiosis and cryptosporidiosis) between calves	Yes, up to 3 weeks
**F2**	AB, 350, conventional	Pens, new calf buildings	6 at first, 12 when drinking milk well, 20 pre-weaning	Fresh bedding daily, mucked out as required according to atmosphere, disinfected and rested between blocks	Yes, below 10˚C
**F3**	AYR, 350, conventional	Hutches	Individual, paired when drinking milk well, 6 pre-weaning	Not covered	Yes, for first week
**F4**	AYR, 120, conventional	Hutches, overflow	Groups of 5	Regularly cleaned, especially the hutches because otherwise ventilation is restricted	Some, for poorly calves

		buildings (old)			
**F5**	AB/SB, 70, conventional	Pens, recent calf sheds (cheap to build)	Individual, groups of 3-4 when drinking well, bigger groups post-weaning	Not covered	No, would consider
**F6**	SB, 300, organic	Hutches	Individual for 2-3 days then groups of 12	Plenty of fresh straw. Mucked -out and disinfected between batches	Some
**F7**	AYR, 280, conventional	Hutches	Individual until pre-weaning groups of 4	Fresh bedding every other day	No
**F8**	Dairy bull calf rearer, batches of 20 calves	Pens	Groups of 20 on automated feeder	Mucked out at group movements	Some, trial with one batch
**F9**	AYR, 250, conventional	Hutches, then pens	Individual hutches, grouped in pens at 2-3 weeks	Cleaned and disinfected (using Bi-OO-Cyst® disinfectant, effective against endoparasites including coccidiosis) between calves	Yes, until moved inside
**F10**	AB, 90, conventional	Pens	Groups of 4-5	Regularly cleaned out with fresh bedding, power wash and disinfect after every calf	Yes, up to 4 weeks
**F11**	AYR, 400, conventional	Pens	Grouped at 5-6 days old, automated feeder	Not covered	Yes, up to 3 weeks
**F12**	AB, 370, conventional	Pens, then outdoors	Groups of 10 until 6 weeks when combined into group of 40	Disinfected and rested between blocks	No, would consider
**F13**	SB, 600, conventional	Pens, then outdoors	Pairs until drinking well, then groups of 15-20, then 35	Pressure wash, quicklime, open doors in summer, let it dry and rested between blocks	No

**F14**	AB, 420, organic	Pens	Individual until 10-14 days old, then groups of 8	Mucked out and disinfected when calves move. Pressure washed and disinfected between blocks	One, for ill calves
**F15**	AYR, 120, conventional	Pens	Groups	Pressure washed	Not covered
**F16**	SB, 250, organic	Pens followed by outdoor paddock	Groups	Mucked out when calves move outside, accommodation rested between calf groups	Not covered
**F17**	Dairy bull/beef calf rearer, 1400 calf places	Pens	Groups	Mucked out, pressure washed, disinfected, rested for about 1 week between batches	No
**F18**	AYR, 180, conventional	Pens	Individual then groups of 5 at 6-7 weeks for weaning	Disinfect for coccidiosis	Yes
**F19**	AYR, 160, conventional	Pens	Not covered	Fresh bedding often, cleaned out, sprayed with peracetic acid, left to dry, rested 1 day between calves	Not covered
**F20**	AB, 330, conventional	Pens	Groups of 4-6, depending on feeder	Steam cleaned and disinfected between every group	Some, for ill calves
**F21**	AYR, 1200, conventional	Pens	Individual until 10 days	Steam cleaned and disinfected	Yes
**F22**	AYR, 130, conventional	Pens	Individual until eating enough	Mucked out once a month, never disinfected	No
**F23**	AB, 250, organic	Pens	Groups of 5, post-weaning groups of 30	Fresh bedding 2-3 times per week, pressure washed, steam cleaned, disinfected	Has some, rarely used
**F24**	AYR, 200, conventional	Pens	Individual first week then moved into groups	Mucked out at group move	Not covered

**F25**	AYR, 350, organic	Hutches, pens in buildings	Groups of 5	Hutches disinfected after every batch of calves. Buildings infrequently disinfected	Trialled for first 4 weeks
**F26**	AB, 500, conventional	Hutches	Groups of 15	Fresh bedding daily, steam cleaned, open and aired/rested between blocks	Not covered

Table content is not exhaustive. Other practices may not have been covered during the interview.
Hutches were located outdoors, pens were inside a building.
